# A trauma‐informed approach to type 1 diabetes mellitus in adults

**DOI:** 10.1111/dme.15510

**Published:** 2025-01-13

**Authors:** Hadassah Buechner, Shreena Unadkat, Joanne Skeldon, Gregory C. Jones

**Affiliations:** ^1^ College of Medical, Veterinary and Life Sciences University of Glasgow Glasgow UK; ^2^ NHS Greater Glasgow and Clyde Glasgow UK

**Keywords:** adverse childhood experiences, psychosocial stress, trauma‐informed, type 1 diabetes mellitus

## Abstract

Suggested mechanisms for an association between early life adversity and worse glycaemic control.

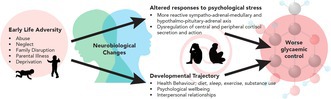

There is a significant body of literature examining psychosocial factors such as adverse life events affecting diabetes monitoring and management in children and adolescents, but very little published in adults. Recent work by Kelly et al. 2023^1^ highlights the burden diabetes has on the psychosocial health of patients, with only 43% of respondents reporting that their diabetes team has ever asked them about their mental well‐being. This commentary aims to synthesise what is known about trauma in adults with type 1 diabetes (T1DM) to better facilitate a move to person‐centred care.

## 1 WHAT IS TRAUMA?

Adverse experiences in childhood or later life, such as physical abuse, sexual abuse, emotional abuse, neglect, or life‐threatening illness, have been well studied under the umbrella term of trauma or toxic stress. Medical literature describes the concept of an allostatic load which links early negative childhood experiences to worse health outcomes, such as cardiovascular disease, asthma and cancers, sometimes called ‘biological embedding’.^2^ Trauma during early life is associated with worse mental health, substance misuse and suicidality,^3^ as well as poor glycaemic control.^4^ Trauma may be associated with the clinical course of Type 1 Diabetes Mellitus directly or indirectly and remains a clinically significant factor in engagement with monitoring and self‐management.

## 2 THE EFFECTS OF EARLY LIFE ADVERSITY ON PATIENTS WITH T1DM

A review of the literature by Franc et al. (2023)^5^ summarised that patients with chronic psychosocial stress were likely to have worsened glycaemic control, as measured by HbA1c and continuous blood glucose monitoring studies. Changes in neurohormonal mechanisms, such as chronic activation of the hypothalamic–pituitary–adrenal (HPA) system and increased sensitivity of the sympathetic‐adrenal‐medullary (SAM) system, lead to a state where hormones such as adrenaline, cortisol and ghrelin raise blood glucose higher than for a given stimulus in a less stressed person.^5^

Early life trauma has been associated with aberrant stress reactivity due to changes in neurodevelopmental pathways, potential dysbiosis affecting the gut‐brain axis and a general pro‐inflammatory state due to changes in epigenetics.^2^ From this research we can extrapolate that people with significant historical or ongoing trauma are likely to experience worsened glycaemic control due to being more reactive to stress.

Furthermore, people who have experienced trauma may present with challenging behaviours associated with mental illness such as depression, anorexia nervosa, complex post‐traumatic stress disorder (C‐PTSD), personality disorders or substance misuse disorders.^3^ Comorbidity with mental health conditions affects compliance with monitoring and management of T1DM.^6^ The overlap between the patient's psychological state and their physical well‐being could manifest as diabulimia,^7^ but may range from not attending routine appointments to failure to follow insulin therapy regimes. Practical approaches to caring for patients with trauma history will be described at the bottom of this commentary.

## 3 STRESSORS ASSOCIATED WITH T1DM DIAGNOSIS AND MANAGEMENT

Diabetes distress has been described as the negative impact that diabetes has on well‐being due to incompatibility with glucose monitoring and the workplace or school, fear of low blood sugars, the effect of diabetes on relationships or barriers to self‐care practices such as exercise.^8^ This distress may be accentuated by existing biopsychosocial difficulties relating to adversity.^2^ Additionally, hospital admissions for diabetic ketoacidosis are a significant psychological stressor and have been linked to an increase in suicidal or self‐injurious behaviour after the episode of ketoacidosis.^9^ People with diabetes report worse psychological well‐being than the average population and 81% felt that psychological care should be routinely available.^1^

Type 1 diabetes mellitus is a condition which may run in families. Early life adversity arguably continues through generations, potentially driven by maladaptive parenting, parental mental illness, and socio‐economic status.^10^ This implies that for people having experienced or currently experiencing adversity, these risk factors may continue to co‐exist with type 1 diabetes in offspring or the patient's parents and extended family. A holistic approach which considers the broader social context should be taken when caring for a patient with trauma.

### 3.1 Trauma‐informed practical guide

A trauma‐informed approach to services involves increasing awareness of the impact of trauma and a commitment to the prevention of traumatisation.

#### 3.1.1 Awareness and education

**FIGURE 1:**
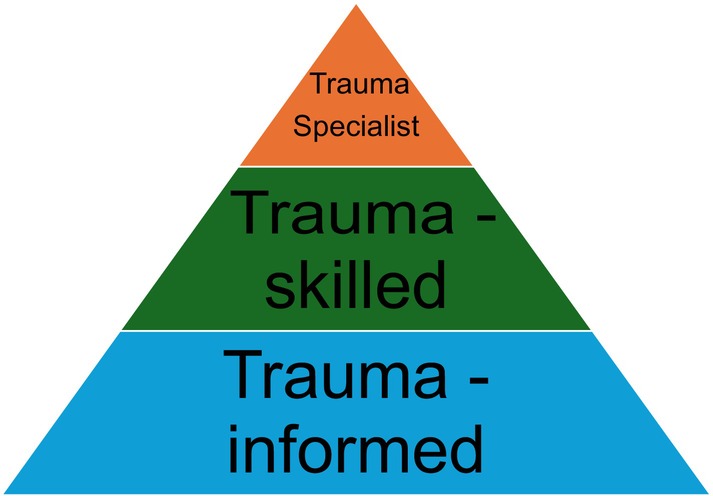
Hierarchy of cumulative training and experience in trauma.

The first step to change is growing awareness of the causes and impact of trauma. Depending on the location and field of work, the application of trauma‐informed practice may vary, but the principles remain the same. A key principle is the concept of different levels of education, training and experience. As laid out in Figure 1, individuals may be trauma‐informed, skilled or specialised.^11^ Depending on a clinician's role and level of exposure to individuals with trauma, different levels of training are required. A diabetologist is likely to fall into either the informed or skilled category, depending on their patient population. ‘Trauma‐informed’ encapsulates awareness and knowledge of trauma and re‐traumatisation, but the clinician is not responsible for delivering interventions or performing detailed assessments relating to trauma.

Another principle of trauma‐informed practice is an awareness that anyone may have been or can be impacted by trauma, including people working inside the organisation, such as clinicians themselves. Resources provided by a given organisation for their employees should acknowledge and offer means to address concerns raised by training materials. Occupational Health services offer a range of well‐being and counselling services for staff and clinicians who have experienced trauma should seek support if required.

#### 3.1.2 Trauma‐informed environments, systems and workforces

While the awareness of trauma and the impact of this on healthcare is ‘everybody's business’,^11^ it is important to note that a systems and environmental approach is required before a patient enters a medical appointment. Managers should be trained in trauma‐informed leadership, relevant to their role in the organisation. Leadership teams should appraise policy and processes which reduce individual choice or have the potential to breach trust (for example safeguarding referrals). The language used in appointments and clinical letters is important to reduce stigma or blame around difficulties with engaging with services. A review of how a given medical environment may contribute to re‐traumatisation could also be required. Services should be aware that those affected by trauma may be most likely to need to develop trust in relationships with health professionals to access the care, treatments and interventions they require. If aspects of the service environment trigger traumatic memories, those affected by trauma may be less likely to seek and engage in support. Potentially triggering situations and environments may include hospital admissions, breaking bad news and painful procedures.^12^ A review of the environmental factors in diabetes care is needed for a trauma‐informed approach. This prevents the entire responsibility for trauma being placed on individual clinicians, which can contribute to workforce burnout^13^ or a reticence to approach the topic with patients due to a lack of systemic support.

#### 3.1.3 Open, holistic conversations about patient well‐being and mental health

People should receive a holistic assessment of the biopsychosocial factors that influence diabetes management, including open conversations about wider aspects of well‐being and the possibility of adverse childhood experiences (ACEs). While there may be concerns that opening up conversations about wider well‐being may exacerbate difficulties,^14^ many people with diabetes reported that asking about their mental well‐being would be an improvement to current services.^1^ Open questions about a person's well‐being, stress levels and personal life allow the patient to control which information they choose to share with their diabetologist, whie setting a tone of understanding. A formalised screening for ACEs may cause distress, and feel intrusive and the clinician may feel inadequately trained to support acute distress arising from direct questioning.^14^ Staff should be empowered to start these conversations by clear signposting pathways to local services, community organisations, and psychoeducational resources.^15^, ^16^

#### 3.1.4 Longer consultations, more frequent clinic reviews or specialist nurse appointments

People who have experienced trauma are likely to need greater support and flexibility from services than typically allocated. Additionally, safety and trust in relationships with staff is a key factor in trauma‐informed practice. People struggling to engage with services or to follow their diabetes treatments should be approached with an attitude of curiosity and an awareness of how trauma may factor into the presentation. Practical solutions to reducing potential barriers such as booking patients in for double‐length appointments, encouraging attendance of a partner or family member or organising more frequent reviews in the community may allow people to engage with services effectively.

#### 3.1.5 Referral to psychology or mental health services

Where available, a referral to psychology or community mental health services should be considered. In the UK most adult diabetes services do not have integrated mental health support. Nevertheless, community mental health services offer trauma‐focused interventions. Despite these services being stretched, a discussion of a mental health referral with the patient would be appropriate.

Additionally, an organisation's psychology service can provide consultation/formulation for teams around complex patients, even when direct intervention is not suitable or required. When considering referral, it is good practice to consider the aims of psychological intervention. This may include input for mental health risk, crisis management, or longer‐term work around self‐management, diabetes‐related distress, and the impacts of early life adversity.
